# The Praise of Fools

**Published:** 1916-07-29

**Authors:** 


### THE PRAISE OF FOOLS.

If it be difficult to suffer with equanimity the strictures of ignorance, praise from such a source is still less tolerable. The events of the hour have provided an abundant supply of both commodities, and many of our adverbs of quality are well-nigh worked to death. The soldiers, the sailors, and the politicians have naturally been the most frequent victims of this gush of superlatives, but others, also, have had their share. The doctors, of course, have not escaped. It is easy to superimpose the marvellous on medicine and to manufacture a miracle out of a commonplace. "Wonderful" cures are almost a stock property even in times of peace, and the war has multiplied opportunities for their production. There is little or no room for the good or efficient or prompt or energetic. The marvellous, the wonderful, and the miraculous are the qualities which are discovered by our critics. Fortunately for the most part individuals have been spared, and it is hospitals and organisations which have been loaded with ignorant and extravagant compliments. We are quite willing to take our meed of praise even as our neighbours, but a little discrimination in the process would not be unwelcome. Experience has taught us that the columns which are crowded with compliments to-day may to-morrow be vehicles for equally meaningless abuse. How certain things get into the papers may have surprised Mr. Crummles, but the avenues are fairly well known nowadays. So long as over-statement is confined to technical matters which are out of the range of the ordinary readers it does little or no harm. The simple gaze and gape and exhaust their scanty stock of exclamations. There is one more passing sensation in a prosaic world, and if heads are temporarily turned inertia soon restores the dull level. Occasionally, however, exaggeration touches a more homely theme and makes mischief in practical fashion. A recent example affords an effective illustration. Workers in munitions, like workers in other things, run a certain amount of risk. Men and women in hundreds and thousands have taken this risk, such as it is, and their zeal and patriotism are beyond reproach. But this recognition is not sufficient for sensational purposes. Hence we are told that numbers of such workers are daily risking their health, undermining their constitution, and voluntarily suffering unsightly disfigurement under the pressure of heroic and self-sacrificing devotion. As these fall smitten out of the ranks others, knowing what fate has in store for them, wait ready for the vacant places. Here is the kind of praise which is capable of working harm. The whole question has been investigated by the medical advisers of the Home Office, who recognise that high explosives do sometimes cause skin irritation, which, however, is not a danger to life and does not persist when the work is discontinued or changed. The workers deserve a full meed of praise, but they may pray to be delivered from the extravagance of their newspaper friends.

